# A multi-modal attention neural network for traffic flow prediction by capturing long-short term sequence correlation

**DOI:** 10.1038/s41598-023-48579-3

**Published:** 2023-12-09

**Authors:** Xiaohui Huang, Yuan Jiang, Junyang Wang, Yuanchun Lan, Huapeng Chen

**Affiliations:** 1 School of Information Engineering, East China Jiaotong University, Nanchang, 330200 China; 2 School of Civil Engineering, East China Jiaotong University, Nanchang, 330200 China

**Keywords:** Computer science, Statistics

## Abstract

Accurate traffic flow prediction information can help traffic managers and drivers make more rational decisions and choices. To make an effective and accurate traffic flow prediction, we need to consider not only the spatio-temporal dependencies between data, but also the temporal correlation between data. However, most existing methods only consider temporal continuity and ignore temporal correlation. In this paper, we propose a multi-modal attention neural network for traffic flow prediction by capturing long-short term sequence correlation (LSTSC). In the model, we employed attention mechanisms to capture the spatio-temporal correlations of the sequences, and the model based on multiple decision forms demonstrated higher accuracy and reliability. The superiority of the model is demonstrated on two datasets, PeMS08 and PeMSD7(M), particularly for long-term predictions.

## Introduction

Over the past few decades, an increasing number of private cars have brought a series of problems such as traffic congestion and parking difficulties. Accurate traffic prediction can provide powerful decision-making basis for traffic managers and enable drivers to choose smoother roads for travel^1^. Traditional machine learning to is often used predict traffic flow, such as Cai et al.^2^ proposed k-nearestneighbor (KNN) for traffic flow prediction. However, the KNN algorithm is a distance-based method that assumes linear relationships between samples. In traffic flow prediction, the relationship between the past and future flow is often nonlinear, making the KNN algorithm less suitable for effectively fitting the data. With the development of deep neural networks, methods such as long short-term memory (LSTM) and gated recurrent unit(GRU) have emerged to handle temporal dependency^3,4^, while methods such as convolutional neural network (CNN) and graph convolution network (GCN) have emerged to handle spatial dependency^5,6^. And some other approaches, such as Medrano et al.^7^ using attention mechanisms and Zhang et al.^8^ leveraging graph convolution, have been effective in predicting traffic flow. However, these prediction methods still have three limitations.

### Fixed spatial dependency

**Figure 1:**
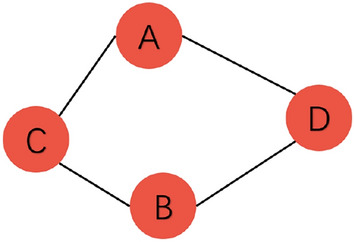
Dynamic spatial dependency.

In the traffic road network, the traffic flow between different nodes often affects and correlates with each other. As shown in Fig. 1, the traffic flow at node *A* may be influenced by nodes *C* and *D*. This influence may even change over time, for example, the correlation between an industrial zone and a residential area may be stronger on workdays but weaker on non-workdays. Therefore, when conducting traffic flow prediction, it is necessary to capture this dynamic spatial dependency.

### Limited-range temporal dependency

**Figure 2:**
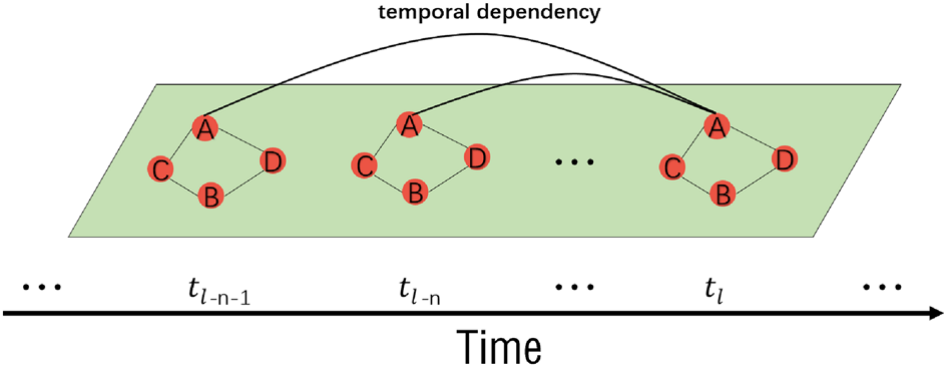
Long temporal dependency.

For a certain node, different historical traffic flows may have different impacts on the current traffic flow at that node. As shown in Fig. 2, the traffic flow at node *A* at time $t_l$ may have weak dependency with the traffic flow at time $t_{l-n}$, but strong dependency with the traffic flow at time $t_{l-n-1}$. Therefore, capturing this type of nonlinear and highly dynamic long temporal dependency is also one of the key points of traffic flow prediction.

### Temporal dependency

**Figure 3:**
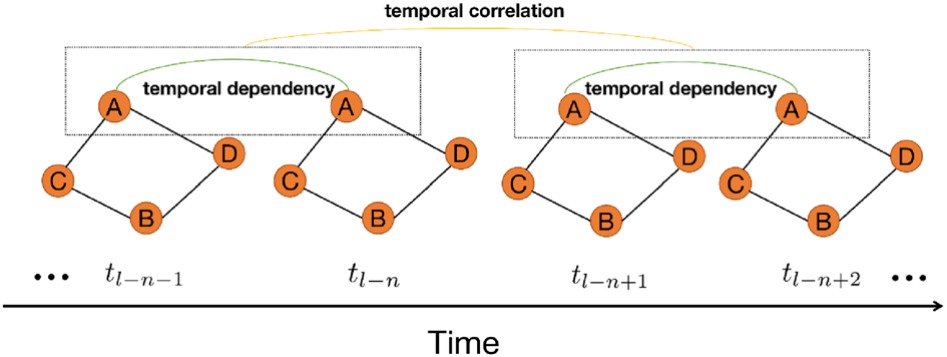
Temporal correlation.

As shown in Fig. 3, temporal dependency refers to the relationship between data at adjacent time points in the time dimension. It describes the relationship between the data at a time point and the data at its adjacent previous or next time point. Temporal correlation refers to the relationship between data in two adjacent time periods in the time dimension. Each time period can contain data from one or more time points, and time correlation describes the mutual association between the data in these two adjacent time periods. This association may be positive, negative, or unrelated, depending on how the data changes over time. If we only consider the time dependency and ignore the correlation between future and past traffic flow, we may not be able to fully utilize the potential information of the traffic flow data. This is because traffic flow often has certain patterns and regularities, such as the flow might change regularly within specific time periods. Therefore, considering the correlation between traffic flow data is very important for improving the accuracy of prediction. This is why combining the time dependency and the correlation between traffic flows in the model helps to improve the prediction accuracy.

In the past few years, with the strong emergence of attention mechanisms in image and natural language processing^9,10^, and the superiority of CNN on Euclidean structured data, we were inspired to apply attention mechanisms and CNN in our model to address spatio-temporal dependencies and temporal correlation.

In this article, we input all traffic flow data into a spatio-temporal feature extraction module to obtain dynamic spatial dependency and long-term temporal dependency. Then, the CNN is used to capture the temporal correlations in the historical traffic flow data and combine it with the spatiotemporal information extracted from the data. An attention-based periodicity module is added to improve the errors caused by a single decision. In summary, the proposed model in this article effectively integrates dynamic spatial dependency, long-term temporal dependency, temporal correlations, and periodicity to enhance the prediction accuracy of traffic flow data. The experimental results demonstrate that incorporating temporal correlations can improve the predictive accuracy of the model. The main contributions of this work can be summarized as follows:

- We propose a capturing long-short term sequence correlation method for discovering the relationship between traffic flow of neighbor time spans.
- We develop a multi-modal attention framework by fusing the periodicity and temporal sequence correlation for traffic flow prediction.
- We evaluate the LSTSC model on two real-world datasets and the experimental results demonstrate that the LSTSC model outperforms the baseline algorithms.

The structure of this work is summarized as follows. “Related work section” section give the related work on traffic flow prediction. The definition and notation of traffic flow have been given in “Definition and notation” section. The general framework of the proposed model is presented in “A multi-modal attention neural network” section. “Experiment and result analysis” section presents the results of the model. Finally, the paper is concluded in “Conclusion” section.

## Related work

In the section, we will elaborate on the traffic flow prediction method based on graph convolution, the methods based on CNN and the methods based on attention mechanism.

### Traffic prediction methods based on CNN

CNN model is one of the most important classical structures in deep learning models, and which is often used to solve the traffic prediction problem. Yang et al.^11^ classified traffic data according to proximity (short-term characteristics), periodicity and trend (long-term characteristics), and mapped them into a two-dimensional space composed of time and space. The high-level spatio-temporal features learned by CNN from matrices with different time lags are further fused with external factors through a logistic regression layer to obtain the final prediction. Zhang et al.^12^ used the spatio-temporal feature selection algorithm (STFSA) to determine the optimal input data time delay and spatial data amount, and extracted the selected spatio-temporal traffic flow features from the actual data and converted them into a two-dimensional matrix. A CNN is later used to learn these features to build a prediction model. Cao et al.^13^ proposed a traffic speed prediction model based on CNN and LSTM. Firstly, CNN was used to extract the daily periodicity and weekly periodicity characteristics of traffic speed in the target area, and the spatio-temporal characteristics of CNN output were extracted through the LSTM layer. Ma et al.^14^ used the nonlinear fitting ability of CNN to extract deep features from the convolutional layer and pooling layer for model training. Yu et al.^15^ used 3D convolutional kernels to simultaneously extract and fuse spatio-temporal features in traffic flow data to ensure that temporal information is treated as spatial information in all network layers.

Although CNN can capture the spatial dependencies in traffic flow prediction, the topology of traffic networks is typically irregular, and traditional CNN are better suited for regular grid-like data, making it challenging to handle irregular data.

### Traffic prediction methods based on graph neural networks

The GCN model acts as a feature extractor just like a CNN, except it works on graphs. Zhao et al.^16^ proposed a temporal GCN, which combined GCN and GRU. In simple terms, for complex topologies of traffic data, we can use GCN to capture spatial dependency and GRU to capture temporal dependency of traffic data. Ali et al.^17^ combined GCN based on LSTM with previously published models to capture spatial patterns and short-time temporal features of images. Chen et al.^18^ proposed a novel location-graph convolutional network (Location-GCN). Location-GCN adds a new learnable matrix to the GCN mechanism, and uses the absolute value of the matrix to represent the different degree of influence between different nodes. Peng et al.^19^ used the dynamic traffic flow probability graph to model the traffic network, and performed graph convolution on the dynamic graph to learn the spatial features of the data, and combined with the LSTM unit to learn the temporal features of the data. Tang et al.^20^ adjusted the graph convolutional network based on spatial correlation to extract the spatial features of the road network.

LSTM was designed to address the issue of short-term time dependencies in traditional RNN. However, in excessively long sequences, problems of gradient vanishing or exploding can still arise. Gradient vanishing prevents the model from learning long-term dependencies, while gradient exploding leads to numerical overflow, causing instability in network training.

### Traffic prediction methods based on attention mechanism

Attention mechanism is a commonly used module in deep learning. As a resource allocation scheme, it uses limited computing resources to process more important information, which is the main means to solve the problem of information overload. Liao et al.^21^ proposed an improved dynamic Chebyshev GCN model. In this method, an attention mechanism based Laplacian matrix update method is proposed, which approximately constructs features from data of different periods. Wang et al.^22^ provided a learnable location attention mechanism that can effectively aggregate the information of neighboring roads. Yin et al.^23^ designed an internal attention mechanism to capture the temporal dependency, and in addition used adjacency as a prior to divide the nodes in the road network into different neighborhood sets. In this way, attention can dynamically capture spatial dependency within and between same-order neighborhoods. Zheng et al.^24^ designed a Conv-LSTM model based on attention mechanism. A reasonable attention mechanism was designed in the model to distinguish the importance of different time stream sequences by automatically assigning different weights. Inspired by the role of attention mechanism in regulating information flow, Wei et al.^25^ embedded the attention mechanism into GRU and LSTM recurrent modules in an attempt to focus on the important information of internal features.

Although the introduction of the attention mechanism has addressed some deficiencies in previous traffic flow models, these attention-based models still lack the capture of temporal correlation, meaning they do not capture the association between future and past data. Inspired by these studies, we use attention mechanisms and CNN to capture spatio-temporal dependencies and temporal correlation separately.

## Definition and notation

In this section, we will give some definition and notations related to traffic flow forecasting.

### Temporal dependency and temporal correlation

As shown in Fig. 3, temporal correlation can be defined as: by observing the historical traffic flows in two adjacent time slots and using CNNs to capture the temporal correlations in the data, the equation is as follows:

1 $$\begin{aligned} \begin{aligned} C=Conv\left( X_{t_1{\sim}t_{n} }, X_{t_{1+n}{\sim}t_{2n}} \right) , \end{aligned} \end{aligned}$$

where *Conv* represents CNN, while $X_{t_1{\sim}t_{n} }$ and $X_{t_{1+n}{\sim}t_{2n}}$ represent two adjacent historical traffic flow data segments in the time dimension. In order to comprehensively capture the spatiotemporal information in the data, we introduced the channel dimension *D*. We are able to leverage the traffic flow information from multiple channels, integrating data from different channels to provide more comprehensive and accurate data features as output.

Temporal dependency can be defind as: by observing the continuous historical traffic flows in a time interval and using attention mechanism to capture the temporal dependencies between the data, the equation is as follows:

2 $$\begin{aligned} \begin{aligned} T=Att\left( X_{t_1{\sim}t_{n} } \right) , \end{aligned} \end{aligned}$$

where *Att* represents attention mechanism.

### Traffic flow prediction problem

${T_{w}^{d}}$th, ${T_{w}^{d+3}}$th and ${T_{w}^{d+6}}$th respectively represent the *d*th day, ${(d+3)}$th day and ${(d+6)}$th day of the *w*th week, and ${T_{w+1}^{d}}$ represents the *d*th day of the ${(w+1)}$th week. We collect the traffic flow data $X_{w;t_1\sim t_{2n}}^{d}$, $X_{w;t_1\sim t_{2n}}^{d+3}$ and $X_{w;t_1\sim t_{2n}}^{d+6}$ for time slots $t_{1}\sim t_{2n}$ on the ${T_{w}^{d}}$th day, ${T_{w}^{d+3}}$th day and ${T_{w}^{d+6}}$th day, as well as the traffic flow data $X_{w+1 ; t_{1}{\sim}t_{n}}^{d}$ for time slots $t_{1}\sim t_{n}$ on the ${T_{w+1}^{d}}$th day as historical traffic flow data, and predict the traffic flow data for time slots ${t_{n+1}\sim t_{2n}}$ on the ${T_{w+1}^{d}}$th day. Traffic flow prediction can be simply expressed as follows:

3 $$\begin{aligned} C= & {} M_1\left( X_{w;t_1\sim t_{2n}}^{d}, X_{w;t_1\sim t_{2n}}^{d+3}, X_{w;t_1\sim t_{2n}}^{d+6}\right) , \end{aligned}$$

4 $$\begin{aligned} {\hat{X}}_{w+1; t_{n+1}{\sim}t_{2n}}^{d}= & {} M_2\left( X_{w+1; t_{1}{\sim}t_{n}}^{d}, C,P\right) , \end{aligned}$$

where *C* represents the temporal correlation of historical traffic flow data, ${\hat{X}}_{w+1; t_{n+1}{\sim}t_{2n}}^{d}$ represents predict the traffic flow data for time slots ${t_{n+1}\sim t_{2n}}$ on the ${T_{w+1}^{d}}$th day, *P* represents the periodicity of historical traffic flow data, and $M_1$ and $M_2$ represent respective components of the traffic flow prediction model. The periodicity of *P* refers to the occurrence or variation of similar events, phenomena, or patterns at the same time intervals every week.

## A multi-modal attention neural network

**Figure 4:**
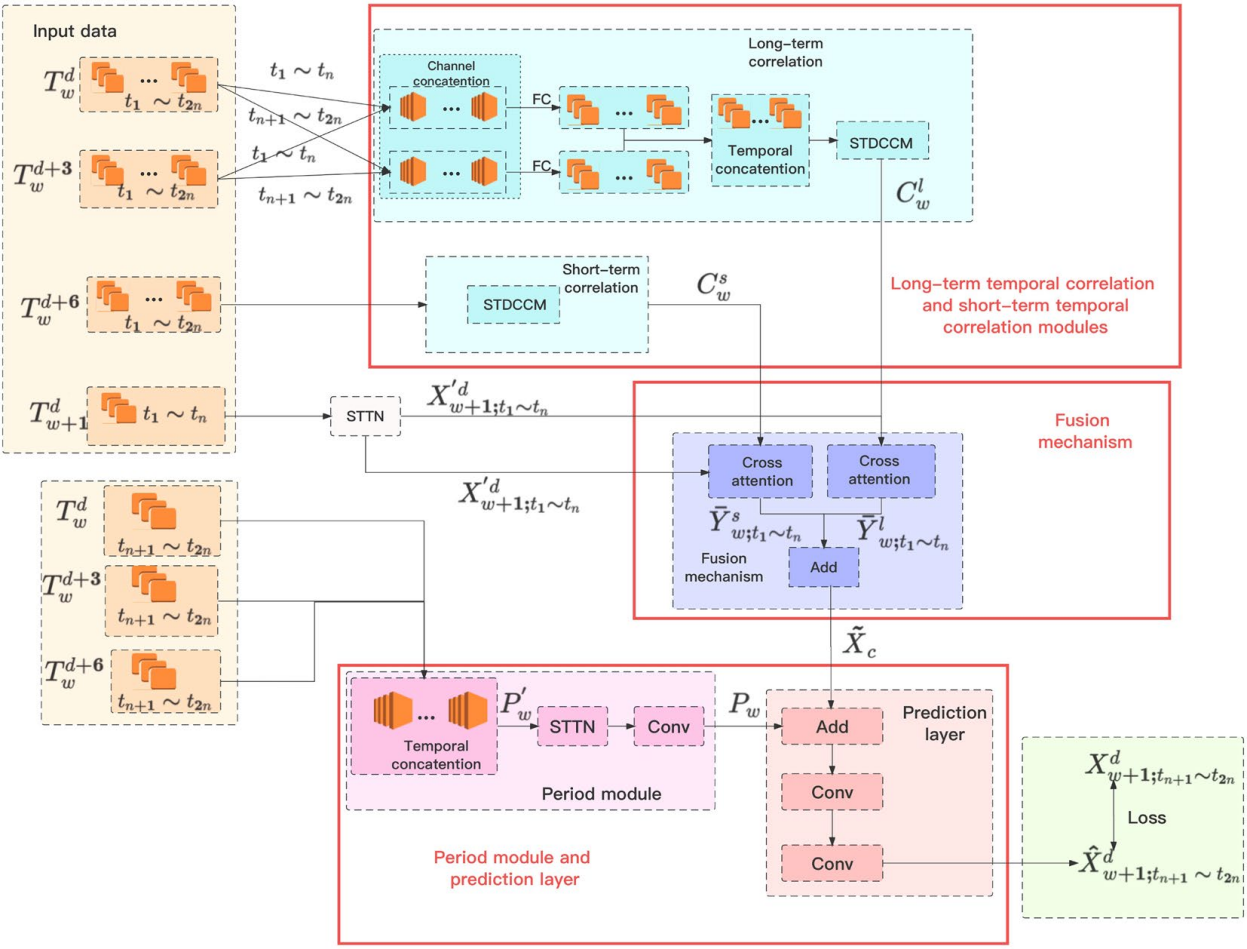
Framework of the LSTSC.

The overall framework of the model is shown in Fig. 4. The model first takes all historical traffic flow data as input, which includes the characteristics of traffic flow in time and space. These data are fed into the (spatial-temporal transformer) STTN module, whose goal is to extract the dynamic spatial dependencies and long-term temporal dependencies from the data. In other words, this module analyzes historical traffic flow data to identify patterns and trends in traffic, which are important information for predicting future traffic flow. Next, the model uses a CNN to continue capturing the long-term and short-term temporal correlation of historical traffic flow data based on spatio-temporal dependencies. By analyzing this data, CNN can identify traffic flow patterns that vary over time, which is crucial for predicting future traffic flow. Then, the model combines the long-term and short-term temporal correlation information extracted by CNN with historical traffic flow data. Finally, the model integrates periodic information into the prediction model to avoid errors caused by single decisions. This is because considering the periodic nature of traffic flow (such as different traffic volumes on weekdays and weekends) is very helpful for improving prediction accuracy.

### Long-term temporal correlation and short-term temporal correlation modules

For the long-term temporal correlation module, the traffic flow data for time slots $t_{1}{\sim}t_{n}$ and $t_{n+1}{\sim}t_{2n}$ on ${T_{w}^{d+3}}$th day and the traffic flow data for time slots $t_{1}{\sim}t_{n}$ and $t_{n+1}{\sim}t_{2n}$ on ${T_{w}^{d+6}}$th day can be spliced on the channel dimension respectively. The equation is as follows:

5 $$\begin{aligned} X_{w; t_{1}{\sim}t_{n}}^{d+3, d+6}= & {} \bigg [X_{w; t_{1}{\sim}t_{n}}^{d+3}, X_{w; t_{1}{\sim}t_{n}}^{d+6}\bigg ], \end{aligned}$$

6 $$\begin{aligned} X_{w; t_{n+1}{\sim} t_{2n}}^{d+3, d+6}= & {} \bigg [X_{w; t_{n+1}{\sim}t_{2n}}^{d+3}, X_{w; t_{n+1}{\sim}t_{2n}}^{d+6}\bigg ], \end{aligned}$$

where the elements inside the brackets [.] are concatenated in a matrix format.

After splicing, the data dimension is reduced through the full connection layer. Finally, the spatio-temporal dependency and correlation capture module (STDCCM) module is input to obtain the spatio-temporal characteristics of the traffic flow data, and the long-term temporal correlation of the data is extracted. For the short-term temporal correlation module, first extract the spatio-temporal characteristics of the traffic flow data for time slots $t_1{\sim}t_{2n}$ on ${T_{w}^{d}}$th day, and finally directly capture the short-term temporal correlation. The reason for the classification of short-term and long-term temporal correlation is that the importance of short-term and long-term temporal correlation may vary in different time steps.

**Figure 5:**
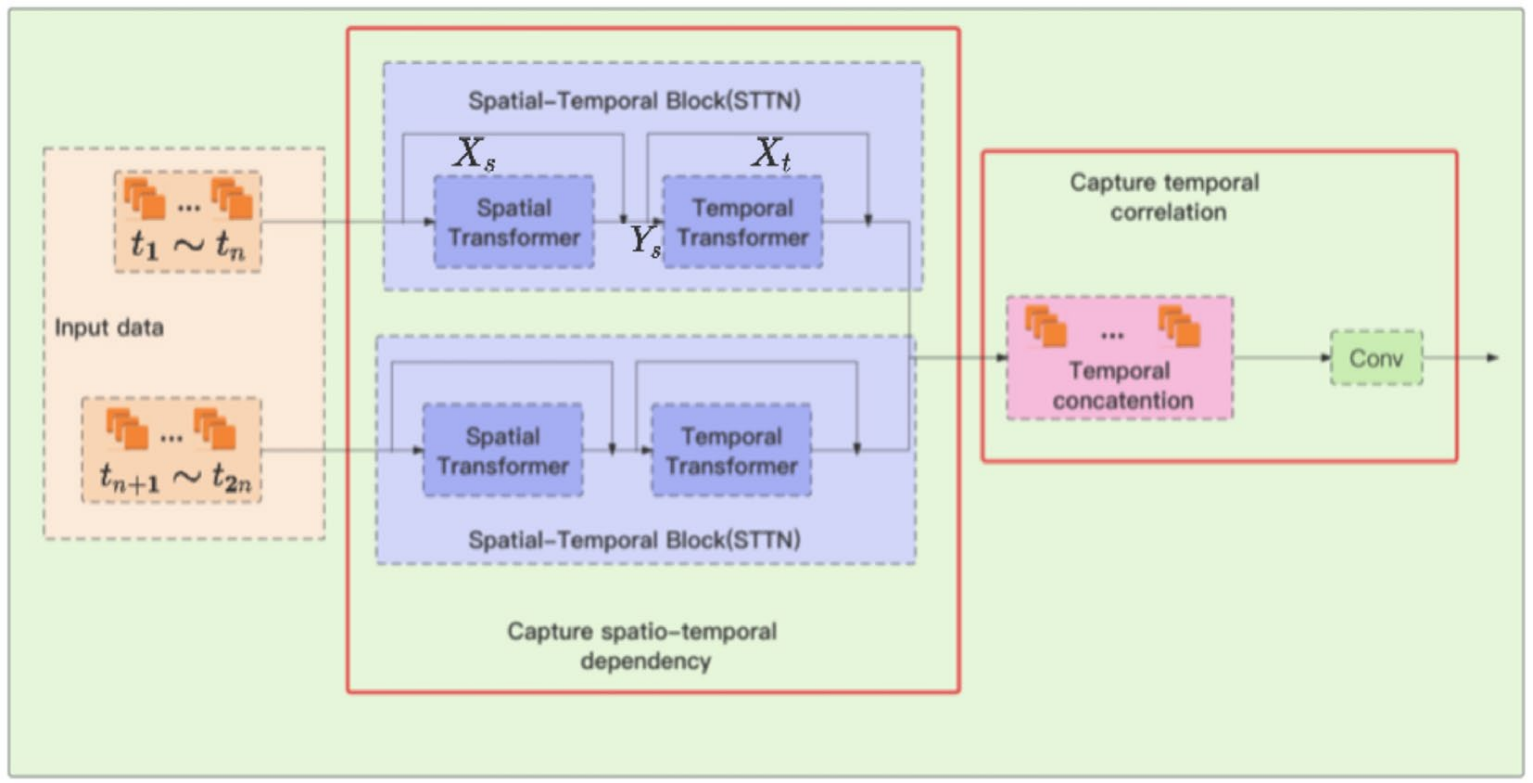
Module structure of STDCCM.

**Figure 6:**
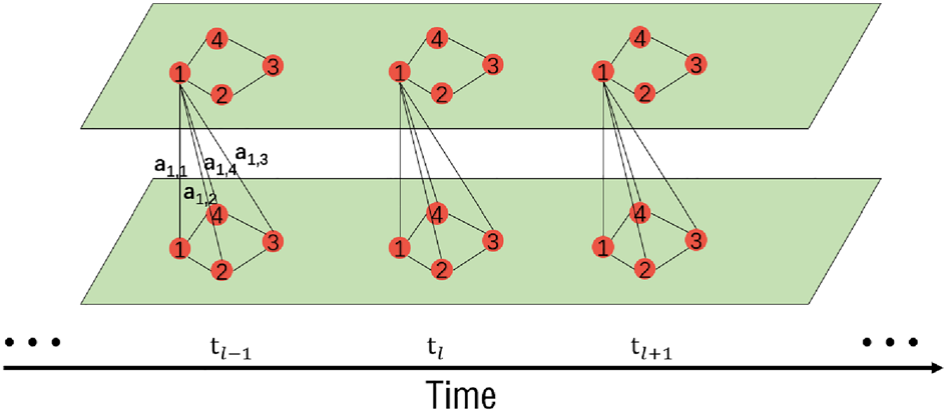
Spatial attention mechanism.

The spatio-temporal dependency and correlation capture module(STDCCM) is shown in Fig. 5. This module consists of two parts, one is the STTN used to extract spatio-temporal dependencies, and the other is used to capture temporal correlation for temporal correlation. It first captures the spatio-temporal features of traffic flow data, and then extracts temporal correlation.

**Figure 7:**
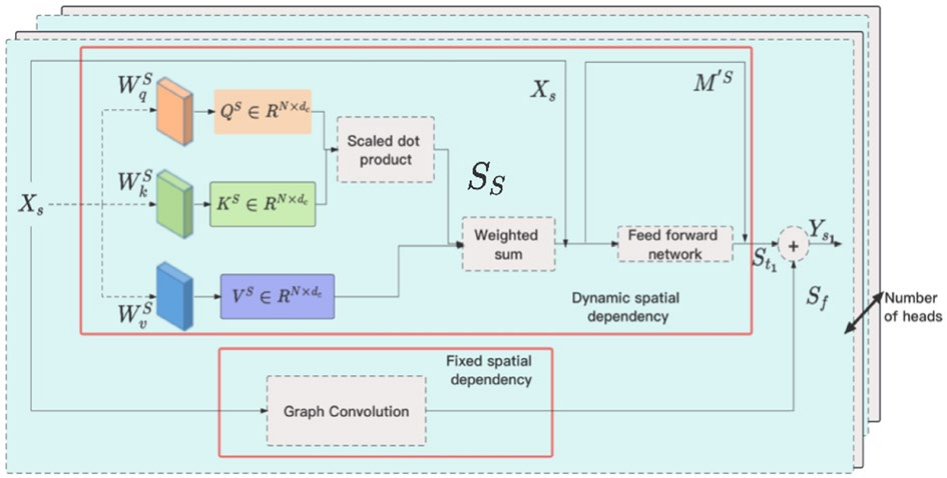
Module structure of spatial transformer.

The STTN is composed of spatial transformer and temporal transformer. The key idea of spatial transformer is to assign different weights to different data points (such as sensors) at different time steps, as shown in Fig. 6, where ${a_{i, j}}$ represents the attention weight between node *i* and node *j* at the same time instant. Spatial transformer is composed of two parts, one is GCN, and the other is attention mechanism, as shown in Fig. 7.

The GCN based on Chebyshev polynomial proximation extracts fixed spatial dependency $S_{f} \in R^{N \times d_{c}}$, where *N* represents the number of sensors.The Chebyshev based graph convolution can effectively leverage the neighborhood information of each node and perform convolution operations on different graph structures, which enhances the performance of GCNs on graph data.

The attention mechanism extracts dynamic spatial dependency $S_{t} \in R^ {N \times d_{c}}$, define three learnable matrices: the query matrix $W_q^S \in R^{d_c \times d_c }$, key matrix $W_k^S \in R^{d_c \times d_c}$, and value matrix $W_v^S \in R^{d_c \times d_c}$. The equations are as follows:

7 $$\begin{aligned} \begin{aligned} {d_c}&= {D}/ {h}, \\ Q^S&= X_sW_q^S, \\ K^S&= X_sW_k^S, \\ V^S&= X_sW_v^S, \\ \end{aligned} \end{aligned}$$

where the query subspace spanned by $Q^S \in R^{N \times d_c}$, the key subspace by $K^S \in R^{N \times d_c}$ and the value subspace by $V^S \in R^{N \times d_c}$. *D* is the channel dimension, and *h* is the number of heads in multi-head attention.

Attention scores $S_S \in R^{N \times N}$ between nodes are calculated with the cross-product of $Q^S$ and $K^S$,

8 $$\begin{aligned} S_S = softmax(Q^S(K^S)^T / \sqrt{{d_c}} ). \end{aligned}$$

Dynamic spatial dependencies $S_{t_1} \in R^{N \times d_c}$ can be obtained based on attention scores, value subspace, and the Residual Network,

9 $$\begin{aligned} \begin{aligned} M^S&= S_S V_S, \\ M^{'S}&= X_s + M^S. \\ \end{aligned} \end{aligned}$$

The inclusion of the feed forward network is to enhance the model’s expressive capacity and non-linear modeling capabilities,

10 $$\begin{aligned} \begin{aligned} U^S&= Relu(Relu(M^{'S}W_0^S)W_1^S)W_2^S, \\ S_{t_1}&= U^S + M^{'S}, \\ \end{aligned} \end{aligned}$$

where $W_0^S$, $W_1^S$, and $W_2^S$ are the weight matrices for the three layers.

The dynamic spatial dependencies and static spatial dependencies are fused using the following equation:

11 $$\begin{aligned} \begin{aligned} {w}&= sigmoid(f_1(S_{f_1})+ f_2(S_{t_1})), \\ Y_{s_1}&= {w}S_{f_1} +(1-{w})S_{t_1}, \\ \end{aligned} \end{aligned}$$

where $f_1$ and $f_2$ represent linear projection to convert $S_{f_1}$ and $S_{t_1}$ into one-dimensional vector.

Finally, the results $Y_s \in R^{N \times D}$ of the multi-head attention mechanism are fused together using the following equation:

12 $$\begin{aligned} Y_s = [Y_{s_1},...,Y_{s_h}]W_3^S + X_s, \end{aligned}$$

where $W_3^S$ is the weight matrix.

Through the multi-head attention mechanism, the model can simultaneously focus on different relationships and patterns, thus better capturing the diversity and complexity in the data. This helps improve the model’s robustness and generalization, making it more effective and flexible in handling various types of input data. Additionally, the multi-head attention mechanism allows the model to attend to different feature interactions at different levels, enabling better extraction of high-level feature representations.

**Figure 8:**
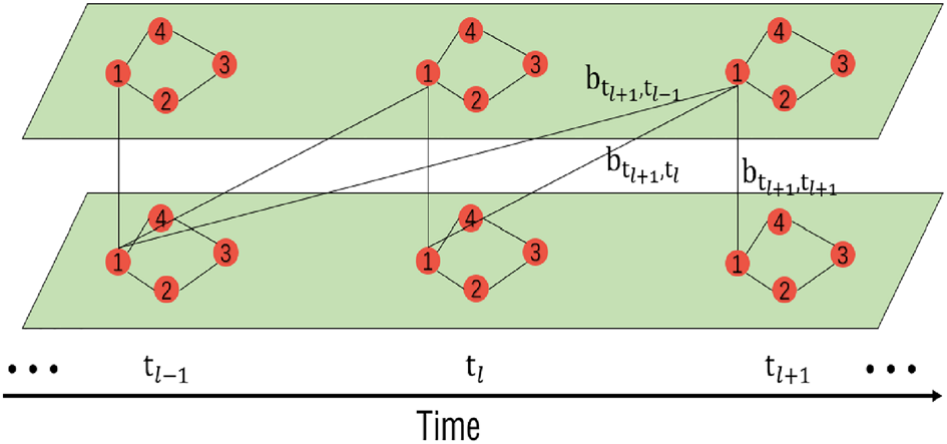
Temporal attention mechanism.

**Figure 9:**
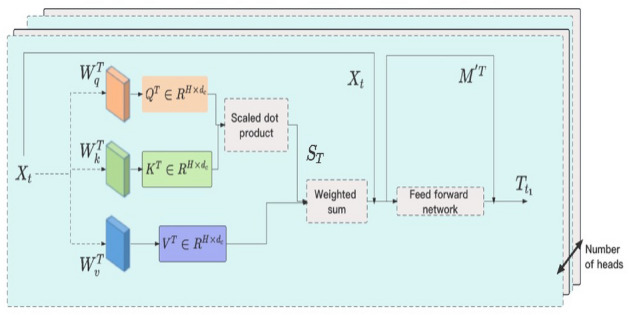
Module structure of temporal transformer.

The key idea of temporal transformer is to achieve the acquisition of temporal dependency by assigning different weights to different time steps, as shown in Fig. 8. ${b_{\alpha , \beta }}$ represents the attention weight, which is the allocation of attention between node 1 at two different time instants. Specifically, if we consider two time instants, such as ${\alpha }$ and ${\beta }$, and a node 1 exists at both time instants, then ${b_{\alpha , \beta }}$ represents the attention weight between the node 1 at time instant ${\alpha }$ and the node 1 at time instant ${\beta }$. Temporal transformer is completely composed of attention mechanism, which can achieve long temporal dependency extraction, as shown in Fig. 9. Here, the value $X_t = Y_s$ that is input to the temporal transformer. Similar to spatial transformer, temporal dependencies are dynamically computed in high-dimensional latent subspaces.

The process of the temporal transformer is similar, with three learnable matrices being defined: the query matrix $W_q^T \in R^{d_c \times d_c }$, key matrix $W_k^T \in R^{d_c \times d_c}$, and value matrix $W_v^T \in R^{d_c \times d_c}$. The equations are as follows:

13 $$\begin{aligned} \begin{aligned} {d_c}&= {D}/ {h}, \\ Q^T&= X_tW_q^T, \\ K^T&= X_tW_k^T, \\ V^T&= X_tW_v^T, \\ \end{aligned} \end{aligned}$$

where the query subspace spanned by $Q^T \in R^{H \times d_c}$, the key subspace by $K^T \in R^{H \times d_c}$ and the value subspace by $V^T \in R^{H \times d_c}$ , where *H* represents the size of the predicted time. *D* is the channel dimension, and *h* is the number of heads in multi-head attention.

Attention scores $S_T \in R^{H \times H}$ between nodes are calculated with the cross-product of $Q^T$ and $K^T$,

14 $$\begin{aligned} S_T = softmax(Q^H(K^H)^T / \sqrt{{d_c}}). \end{aligned}$$

Temporal dependencies $T_{t_1} \in R^{H \times d_c}$ can be obtained based on attention scores, value subspace, and the Residual Network,

15 $$\begin{aligned} \begin{aligned} M^T&= S_T V_T, \\ M^{'T}&= X_t + M^T, \\ U^T&= Relu(Relu(M^{'T}W_0^T)W_1^T)W_2^T, \\ T_{t_1}&= U^T + M^{'T}, \\ \end{aligned} \end{aligned}$$

where $W_0^T$, $W_1^T$, and $W_2^T$ are the weight matrices for the three layers.

Finally, the results $Y_t \in R^{H \times D}$ of the multi-head attention mechanism are fused together using the following equation:

16 $$\begin{aligned} Y_t = [T_{t_1},...,T_{t_h}]W_3^T + X_t, \end{aligned}$$

where $W_3^T$ is the weight matrix.

Temporal correlation is entirely composed of CNN and can capture temporal correlation by first concatenating the traffic flow data on the time dimension and then obtaining the temporal correlation through CNN.

17 $$\begin{aligned} C_w^l= & {} Conv([Y_{t_1}^l, Y_{t_2}^l]), \end{aligned}$$

18 $$\begin{aligned} C_w^s= & {} Conv([Y_{t_1}^s, Y_{t_2}^s]). \end{aligned}$$

$Y_{t_1}^l$ signifies the spatio-temporal dependency of $t_1{\sim}t_n$ within the long-term temporal correlation module. $Y_{t_2}^l$ represents the spatio-temporal dependency of $t_{n+1}{\sim}t_{2n}$ within the long-term temporal correlation module. $Y_{t_1}^s$ corresponds to the spatio-temporal dependency of $t_1{\sim}t_n$ within the short-term temporal correlation module. $Y_{t_2}^s$ indicates the spatio-temporal dependency of $t_{n+1}{\sim}t_{2n}$ within the short-term temporal correlation module.

### Fusion mechanism

**Figure 10:**
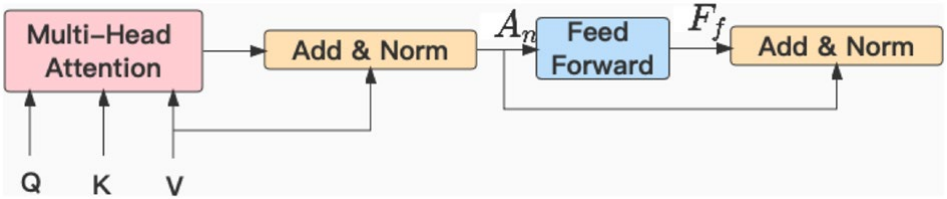
Cross attention structure.

This module is mainly composed of attention mechanism, and its function is to realize the combination of temporal correlation and historical traffic flow data. The module consists of two parts, cross attention and data fusion. The structure of cross attention is shown in Fig. 10. We take the combination of short term temporal correlation and historical traffic flow data as an example, where the query subspace by $Q = Q_{d} \in R^{H \times d_m}$ , the key subspace by $K = K_{d} \in R^{H \times d_m}$ and the value subspace by $V = V_{d} \in R^{H \times d_m}$. The equation is as follows:

19 $$\begin{aligned} \begin{aligned} Q_{d}&=X_{w+1; t_1{\sim}t_{n}}^{\prime d} W_{q}, \\ K_{d}&=C_{w}^{s} W_{k}, \\ V_{d}&=C_{w}^{s} W_{v}, \\ {d_{m}}&={D} / {h}, \\ A_{d}&={softmax}\left( Q_d K_d^{T} / {d_{m}}\right) , \\ A_n&= LayerNorm(A_dV_d + V_d), \\ F_f&= Relu(A_nW_1^F)W_2^F), \\ {\bar{Y}}_{w; t_1{\sim}t_{n}}^{s}&=LayerNorm(F_f + A_n), \end{aligned} \end{aligned}$$

where query matrix $W_{q} \in R^{d_m \times d_m}$, key matrix $W_{k} \in R^{d_m \times d_m}$ and value matrix $W_{v} \in R^{d_m \times d_m}$. They are responsible for converting the data information to the corresponding query subspace $Q_{d}$, the key subspace $K_d$ and the value subspace $V_d$. $W_1^F$ and $W_2^F$ represent weight matrices, and *LayerNorm* refers to layer normalization, which transforms the input of each neuron in a layer to have the same mean and variance, thereby accelerating convergence. *D* is the channel dimension of the data, *h* is the number of multiple attention. The spatio-temporal dependencies were captured by STTN for the traffic flow data in time slots $t_1\sim t_{n}$ on ${T_{w+1 ; t_{1}{\sim}t_{n}}^d}$th day, and this resulted in $X_{w+1 ; t_{1}{\sim}t_{n}}^{\prime d}$. The same process applies to the long term temporal correlation cross attention module.

The calculation equation used in the data fusion module is shown as follows:

20 $$\begin{aligned} {\tilde{X}}_c=W_{s} {\bar{Y}}_{w; t_1{\sim}t_{n}}^{s}+W_{l} {\bar{Y}}_{w; t_1{\sim}t_{n}}^{l}, \end{aligned}$$

where $W_s$, $W_l$ are weight matrices, and ${\bar{Y}}_{w ; t_1{\sim}t_{n}}^{s}$ and ${\bar{Y}}_{w ; t_1{\sim}t_{n}}^{l}$ are the output results of short-term and long-term cross attention, respectively.

### Period module and prediction layer

In order to reduce the error caused by a single decision, a period module is proposed. The module uses the traffic flow data at the time of ${T_w^{d}}$th day, ${T_w^{d+3}}$th day and ${T_w^{d+6}}$th day, and first splices the data on the time dimension to obtain the output $P_{w}^{'} \in R^{3H\times N}$, then extracts the spatio-temporal dependency of the data, and then reduces the dimension through the convolution neural network to obtain the final result $P_{w} \in R^{H\times N}$ of the module, where *H* represents the size of the predicted time, and *N* represents the number of sensors.

Then the output result of the period module is used as the input data of the prediction layer, which is composed of two layers of convolution. The equation is as follows:

21 $$\begin{aligned} {\hat{X}}_{w+1; t_{n+1}{\sim}t_{2n}}^{d}= Conv(Conv( {\tilde{X}}_c + P_w )). \end{aligned}$$

## Experiment and result analysis

In this section, the experimental process is described in detail from the following aspects: datasets, baselines, evaluation metrics, hyperparameter setting, convergence analysis, performance comparison and ablation studies.We use traffic speed data as traffic flow information.

### Datasets

Two real datasets: PeMSD7(M) and PeMS08, are used to evaluate the performance of LSTSC model. All the data is scaled to 0 to 1 with min-max normalization in the experiments, and the details of the datasets are shown in Table 1.

- *PeMSD7(M)* The traffic speed dataset is collected by the California Department of Transportation in the seventh district of California through 228 road traffic sensors, and the collected data samples are aggregated every 5 min. The dataset records the vehicle speed of the seventh district of California from May 1, 2012 to June 30, 2012.
- *PeMS08* The traffic speed dataset is collected by the California Department of Transportation through 170 road traffic sensors, and the collected data samples are aggregated every 5 min. The dataset records the vehicle speed of San Bernardino, California, from July 1, 2016 to August 31, 2016.

**Table 1:** Details of the datasets.

Datasets	PeMSD7(M)	PeMS08
Start time	2012/05/01	2016/07/01
End time	2012/06/30	2016/08/31
Training set	2012/05/01–05/31	2016/07/01–08/01
Validating set	2012/06/01–06/15	2016/08/02–08/16
Testing set	2012/06/16–06/30	2016/08/17–08/31
Time interval	5 (min)	5 (min)

### Baselines

The following provides a description of the baseline algorithms that are compared with the LSTSC model.

- FC-LSTM: As LSTM only considers the time series and does not take into account the spatial correlation between them, FC-LSTM is an improvement of the LSTM model by adding an attention mechanism, where the input of each gate is determined by three parts.
- DCRNN^26^: DCRNN introduces diffusion convolution as graph convolution to capture spatial dependency, and uses sequence-sequence architecture combined with GRU to capture temporal dependency.
- STGCN^27^: STGCN introduces the graph neural network into the prediction of spatio-temporal series to effectively extract the spatio-temporal dependency.
- GWNet^28^: GWNet includes two components, one is the adaptive dependency matrix, which is used to extract spatial dependency, and the other is the stacked dependent 1D conversion, which is used to extract temporal dependency.

### Evaluation metrics

The evaluation metrics of LSTSC model are the same as before^23^, including mean absolute error(MAE), root mean square error(RMSE) and mean absolute percentage error(MAPE). The equation is as follows:

22 $$\begin{aligned} MAE= & {} \frac{1}{n} \sum _{i=1}^{n}\left| y_{i}-{\hat{y}}_{i}\right| , \end{aligned}$$

23 $$\begin{aligned} RMSE= & {} \sqrt{\frac{1}{n} \sum _{i=1}^{n}\left( y_{i}-{\hat{y}}_{i}\right) ^{2}}, \end{aligned}$$

24 $$\begin{aligned} MAPE= & {} \frac{1}{n} \sum _{i=1}^{n}\left| \frac{{\hat{y}}_{i}-y_{i}}{y_{i}}\right| , \end{aligned}$$

where $y_i$ represents the actual value at a certain moment in ${T_{w+1;t_{n+1}{\sim}t_{2n}}^d}$th day, and ${\hat{y}}_{i}$ represents the corresponding predicted value. n represents the size of the predicted time. The reason why the above three metrics are selected in this paper is that MAE and RMSE can better reflect the actual situation of the predicted value error. For MAPE, theoretically, the smaller its value, the better the fitting effect of the prediction model and the better accuracy.

### Parameter settings

Table 2 describes the parameters of LSTSC in the experiment. We use 12 historical time steps to predict the next 12 time steps in the future. The CNN module, designed to extract temporal correlation, consists of a one-layer CNN with 12 filters, a stride of 1, a padding size of 0, and a convolution kernel size of $1 \times 1$. The number of heads for multi-head attention in the experiment is uniformly set to 2. The CNN module used in the prediction layer is a two-layer CNN, with the number of filters set to 12 and 1 respectively, a stride of 1, a padding size of 0, and a convolution kernel size of $1 \times 1$. LSTSC is optimized by Adam optimizer, and the batch size of the experiment is set to 16.

**Table 2:** Hyper parameter settings for the model.

Parameter	Value
CNN layers (STDCCM)	1
CNN layers (prediction layer)	1
Number of filters in CNN (STDCCM)	12
Number of filters in CNN (prediction layer)	12,1
Number of heads in attention	2
Padding in CNN (STDCCM)	0
Stride in CNN (STDCCM)	1
Stride in CNN (prediction layer)	1,1
Padding in CNN (prediction layer)	0,0
Order for Chebyshev polynomials	2
Convolution kernel size	(1,1)
Batch size	16
Times of training (Epoch)	300
Optimizer	Adam
Learning rate	0.05
Dropout	0.2
n	12

### Hyperparametric studies

In this section, we investigate the influence of the dimension $\alpha$ of feed forward network to the results of traffic flow prediction, which belongs to the multi-head attention mechanism. We study the result of traffic flow prediction when $\alpha$ is 1, 2, 3, 4. As shown in Table 3 (the best results in the table have already been indicated in bold.),The best experimental results for the PeMSD7(M) dataset were achieved when $\alpha$ = 2. When using the PeMS08 dataset, the model achieved the best results for the MAE metric at 15 min and 30 min when $\alpha$ = 4, and at 60 min when $\alpha$ = 2. For the MAPE metric, the model achieved the best results at 15 min and 30 min when $\alpha$ = 3, and at 60 min when $\alpha$ = 2. For the RMSE metric, the model achieved the best results at 15 min, 30 min, and 60 min when $\alpha$ = 2. Therefore, when conducting long-term traffic flow forecasting, $\alpha$ value of 2 may be used.

**Table 3:** The traffic flow prediction results with the change of the parameters.

$\alpha$ value (PeMSD7(M))	MAE (15/30/45 min)	MAPE (%) (15/30/45 min)	RMSE (15/30/45 min)
$\alpha$ = 1	2.20/2.84/3.24	5.16/7.05/8.24	4.12/5.51/6.31
$\alpha$ = 2	**2.17/2.75/3.07**	**5.15/6.81/7.75**	**4.05/5.33/6.00**
$\alpha$ = 3	2.20/2.81/3.14	5.21/6.97/8.01	4.15/5.46/6.16
$\alpha$ = 4	2.17/2.78/3.11	5.17/6.89/7.82	4.06/5.36/6.08

$\alpha$ value (PeMS08)	MAE (15/30/60 min)	MAPE (%) (15/30/60 min)	RMSE(15/30/60 min)
$\alpha$ = 1	13.77/14.22/15.54	9.21/9.52/10.57	22.22/23.49/25.96
$\alpha$ = 2	13.54/14.07/ **15.34**	8.96/9.38/**10.45**	**22.02/23.40/25.82**
$\alpha$ = 3	13.51/14.06/15.50	**8.83/9.37** /10.74	22.05/23.42/25.84
$\alpha$ = 4	**13.36/14.01** /15.43	8.85/9.37/10.45	22.03/23.48/26.06

### The convergence of LSTSC model

**Figure 11:**
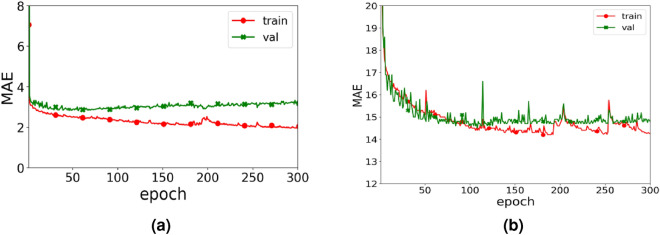
The training and validation results on the two datasets: (**a**) PeMSD7(M) dataset; (**b**) PeMS08 dataset.

Figure 11 shows the loss curve of LSTSC model on two real datasets about training set and verification set during the experiment. By observing Fig. 11(a), we can find that on the PeMSD7(M) dataset, for the training set and the verification set, the MAE of the two datasets gradually decreases with the increase of the number of training iterations, but when the number of iterations is 65, the MAE of the training set and the verification set starts to reach a certain stability. By observing Fig. 11(b), for the training set and verification set of PeMS08 dataset, the MAE of both datasets gradually decreases with the number of training iterations increasing, but when the number of iterations is 128, the MAE of the training set and verification set starts to reach a certain stability.

### Experimental results and analysis

The Highway Capacity Manual^29^ recommends using a 15 min as short-term prediction interval for research and analysis purposes^30^. Table 4 describes the results of LSTSC model and baseline algorithm on PeMSD7(M) and PeMS08 datasets. For the PeMSD7(M) dataset and PeMS08 dataset, the performance of DCRNN and STGCN is better than that of FC-LSTM algorithm, which shows that road network information is crucial for traffic flow prediction. GWNet and LSTSC algorithms are superior to DCRNN and STGCN algorithms, which shows that time information is also essential for accurate prediction of traffic flow. For the PeMSD7(M) dataset, comparing the results of GWNet algorithm and LSTSC algorithm, based on the above three evaluation metrics, LSTSC is not as good as GWNet algorithm in short-term prediction ($\le$ 15 min), but LSTSC is better than GWNet algorithm in medium and long-term prediction (> 15 min). For the PeMS08 dataset, the LSTSC model based on MAE evaluation index is superior to GWNet in both short-term traffic flow prediction and medium and long-term traffic flow prediction. The short-term traffic flow prediction result of LSTSC model based on MAPE evaluation index is not as good as that of GWNet model, but in the face of medium and long-term traffic flow prediction, LSTSC model is better than that of GWNet model. The short-term traffic flow prediction result of LSTSC model based on RMSE evaluation index is not as good as that of GWNet model, but in the face of medium and long-term traffic flow prediction, LSTSC model is better than that of GWNet model. The above results show that strengthening the capture of temporal correlation may help improve the accuracy of medium and long-term traffic flow prediction.

**Table 4:** The experimental results of different models.

Models (PeMSD7(M))	MAE (15/30/45 min)	MAPE (%) (15/30/45 min)	RMSE(15/30/45 min)
FC-LSTM	3.57/3.94/4.16	8.60/9.55/10.10	6.20/7.03/7.51
DCRNN	2.37/3.31/4.01	5.54/8.06/9.99	4.21/5.96/7.13
STGCN	2.25/3.03/3.57	5.26/7.33/8.69	4.04/5.70/6.77
GWNet	**2.14**/2.80/3.19	**4.93**/6.89/8.04	**4.01**/5.48/6.25
LSTSC	2.17/**2.75/3.07**	5.15/**6.81/7.75**	4.05/**5.33/6.00**

Models (PeMS08)	MAE (15/30/60 min)	MAPE (%) (15/30/60 min)	RMSE (15/30/60 min)
FC-LSTM	17.38/21.22/30.69	12.63/17.32/25.72	26.27/31.97/43.96
DCRNN	14.16/15.24/17.70	9.31/9.90/11.13	22.20/24.26/27.14
STGCN	14.95/15.92/17.65	9.87/10.42/11.34	23.48/25.36/28.03
GWNet	13.72/14.67/16.15	**8.80**/9.49/10.74	**21.71**/23.50/25.95
LSTSC	**13.54/14.07/15.34**	8.96/**9.38/10.45**	22.02/**23.40/25.82**

**Figure 12:**
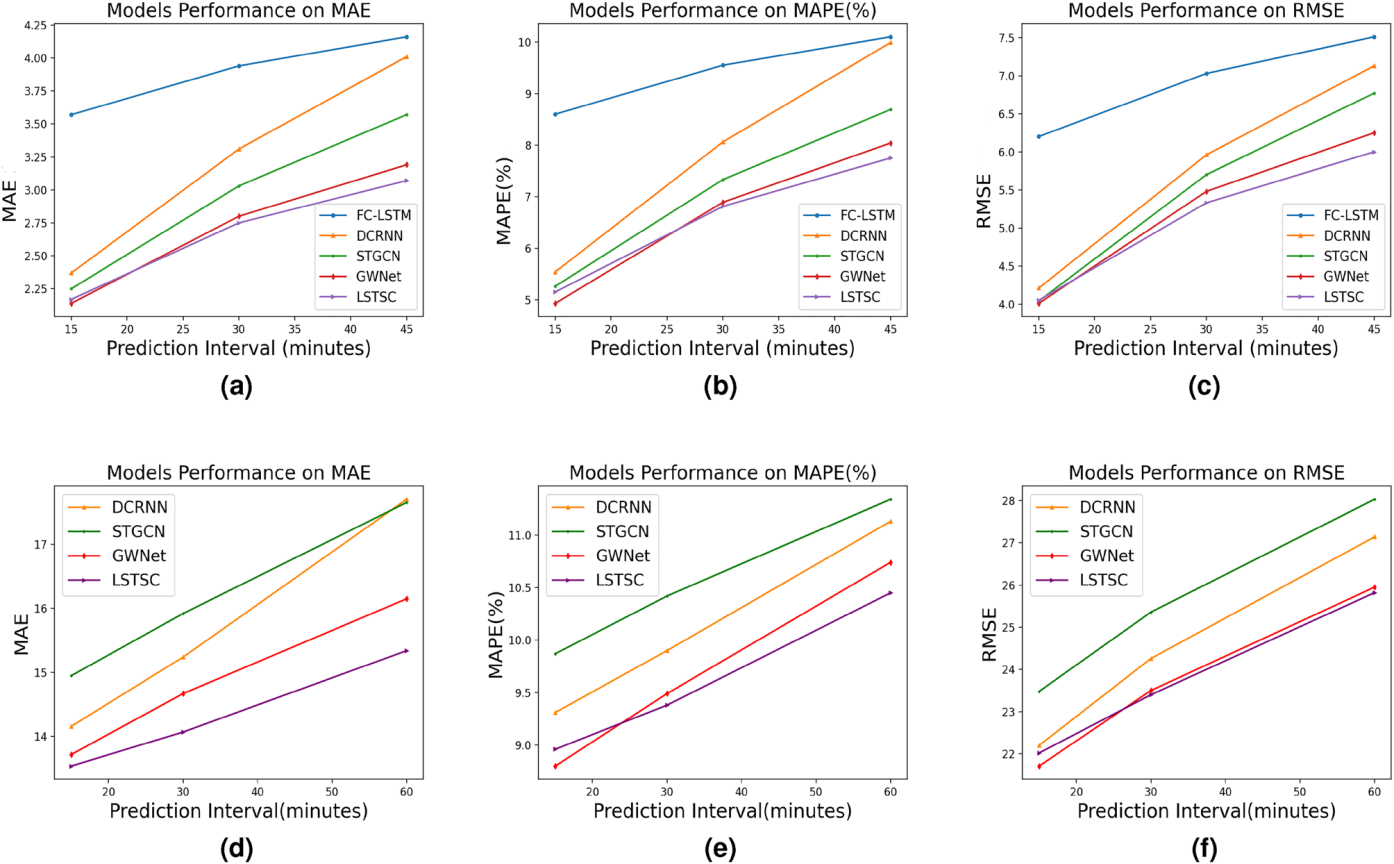
The experimental results on the two datasets: (**a**) MAE evaluation of model performance on PeMSD7(M) dataset; (**b**) MAPE (%) evaluation of model performance on PeMSD7(M) dataset; (**c**) RMSE evaluation of model performance on PeMSD7(M) dataset; (**d**) MAE evaluation of model performance on PeMS08 dataset; (**e**) MAPE (%) evaluation of model performance on PeMS08 dataset; (**f**) RMSE evaluation of model performance on PeMS08 dataset.

In order to observe the changes in evaluation metrics of each model more intuitively, as shown in Fig. 12, based on two real datasets, the rate of increase in MAE, MAPE, and RMSE values of the LSTSC model is lower than the baseline model as the prediction time advances, indicating that the long-term traffic flow prediction values of the LSTSC model are closer to the real values. Therefore, for long-term traffic flow prediction, the LSTSC model has more advantages.

### Ablation studies

In this section, various ablation experiments are used to test the effectiveness of modules on LSTSC. These modules mainly include period module, long-term connection and short-term connection modules. The variants are listed below:

- *LSTSC_ NoL:* Apply short-term temporal correlation and period module to forecast traffic flow.
- *LSTSC_ NoS:* Use long-term temporal correlation and period module to forecast traffic flow.
- *LSTSC_ NoP:* Do not use period module to forecast traffic flow.
- *LSTSC:* The model includes long-term and short-term temporal correlation and non-single decision for traffic flow prediction.

**Table 5:** Comparison of experimental results of model variation.

Varients (PeMSD7(M))	MAE (15/30/45 min)	MAPE (%) (15/30/45 min)	RMSE(15/30/45 min)
LSTSC_NoL	2.25/2.86/3.22	5.30/7.05/8.12	4.17/5.55/6.34
LSTSC_NoS	2.19/2.79/3.13	5.17/7.02/8.09	4.11/5.46/6.17
LSTSC_NoP	2.30/2.92/3.28	5.40/7.24/8.37	4.22/5.64/6.43
LSTSC	**2.17/2.75/3.07**	**5.15/6.81/7.75**	** 4.05/5.33/6.00**

Varients (PeMS08)	MAE (15/30/60 min)	MAPE (%) (15/30/60 min)	RMSE (15/30/60 min)
LSTSC_NoL	13.77/14.31/15.79	9.24/9.61/10.78	22.41/23.72/26.25
LSTSC_NoS	13.68/14.18/15.47	9.16/9.53/10.72	22.31/23.43/25.85
LSTSC_NoP	14.37/14.95/16.37	9.30/9.81/10.80	23.04/24.13/26.68
LSTSC	**13.54/14.07/15.34**	**8.96/9.38/10.45**	**22.02/23.40/25.82**

For the ablation experiment on Eq. (20), we set $W_s$ to a zero matrix while $W_l$ remains a learnable parameter matrix. As a result, the contribution of ${\bar{Y}}_{w; t_1{\sim}t_{n}}^{s}$ to the model output is eliminated, and the importance of ${\bar{Y}}_{w ; t_1{\sim}t_{n}}^{s}$ can be assessed by comparing the model performance before and after ablation. A similar operation is performed for the ablation experiment on ${\bar{Y}}_{w ; t_1{\sim}t_{n}}^{s}$, where $W_l$ is set to a zero matrix and $W_s$ remains a learnable parameter matrix. As a result, the contribution of ${\bar{Y}}_{w; t_1{\sim}t_{n}}^{s}$ to the model output is eliminated. The reason for choosing this method is that we want to ablate the input features without changing the model structure, by merely modifying the weight matrices. By setting the weight matrix of a specific input feature to a zero matrix, we can completely eliminate the contribution of that feature to the model output, thereby assessing the importance of the feature. Additionally, since the result of multiplying any matrix by a zero matrix is still a zero matrix, this method is also computationally efficient.

Table 5 describes the results of the LSTSC model and its variants on the PeMSD7(M) and PeMS08 datasets. According to the experimental results of the two datasets, it can be found that the LSTSC model performs better than the LSTSC_NoLong, LSTSC_NoShort, and LSTSC_NoPeriod models for both short-term and long-term traffic flow prediction, respectively proving the effectiveness of long-term temporal correlation, short-term temporal correlation, and period. For the PeMSD7(M) dataset, the experimental results of LSTSC_NoShort are better than those of LSTSC_NoLong and LSTSC_NoPeriod, indicating that short-term temporal correlation has a lower weight than long-term temporal correlation and period, while the experimental results of LSTSC_NoPeriod are worse than those of LSTSC_NoLong, indicating that the weight of period is higher than that of long-term temporal correlation. For the PeMS08 dataset, the experimental results based on MAE, MAPE and RMSE metrics still reflect the conclusions obtained from the PeMSD7(M) dataset, where short-term temporal correlation have lower weights compared to long-term temporal correlation and periodicity, and periodicity has higher weights compared to long-term temporal correlation.

Due to the inherent periodicity in natural phenomena, traffic flow might exhibit cyclic patterns, with traffic patterns recurring on a weekly basis, for instance. Consequently, long-term temporal correlation could be more pronounced compared to short-term temporal correlation. In other words, traffic patterns may tend to repeat over longer time scales, such as a week, leading to stronger correlations in the long-term compared to short-term correlations. For example, let’s consider a major urban freeway that experiences heavy traffic during weekdays due to work commutes, resulting in a daily traffic pattern. However, on weekends, the traffic flow on the same freeway might decrease significantly, leading to a different traffic pattern. Over time, this daily pattern may not be as consistent as the weekly pattern, where traffic flow experiences regular fluctuations during weekdays and weekends. The long-term temporal correlation, in this case, would capture the recurrent weekly pattern, while the short-term temporal correlation would mainly reflect the daily fluctuations.

In general, the long-term temporal correlation module, short-term temporal correlation module and period module can effectively improve the traffic flow prediction performance of the model.

## Conclusion

In order to strengthen the capture of temporal correlation and effectively solve the dynamic spatial dependency and long-term temporal dependency in traffic flow prediction, we propose a multi-modal attention neural network for traffic flow prediction. In this model, an attention mechanism is designed to address the limited temporal dependency and fixed spatial dependency problems of the data. At the same time, CNNs are used to enhance the capture of temporal correlation in traffic data, and a fusion mechanism is designed to obtain the prediction results. In addition, we also design a multimodal attention neural network to solve the problem of single decision-making in the model. Finally, various experiments were conducted on two real-world datasets, and the results show that the performance of the proposed model in long-term traffic flow prediction is better than that of baseline algorithms.

## Data Availability

The datasets used and analysed during the current study are available from the corresponding author on reasonable request.
